# Genome-wide and Ordered-Subset linkage analyses provide support for autism loci on 17q and 19p with evidence of phenotypic and interlocus genetic correlates

**DOI:** 10.1186/1471-2350-6-1

**Published:** 2005-01-12

**Authors:** Jacob L McCauley, Chun Li, Lan Jiang, Lana M Olson, Genea Crockett, Kimberly Gainer, Susan E Folstein, Jonathan L Haines, James S Sutcliffe

**Affiliations:** 1 Center for Human Genetics Research, Departments of Molecular Physiology & Biophysics, Vanderbilt University, Nashville, TN, USA; 2 Biostatistics, Vanderbilt University, Nashville, TN, USA; 3 Department of Psychiatry and Behavioral Sciences, Johns Hopkins University, Baltimore, MD, USA

## Abstract

**Background:**

Autism is a neurobehavioral spectrum of phenotypes characterized by deficits in the development of language and social relationships and patterns of repetitive, rigid and compulsive behaviors. Twin and family studies point to a significant genetic etiology, and several groups have performed genomic linkage screens to identify susceptibility loci.

**Methods:**

We performed a genome-wide linkage screen in 158 combined Tufts, Vanderbilt and AGRE (Autism Genetics Research Exchange) multiplex autism families using parametric and nonparametric methods with a categorical autism diagnosis to identify loci of main effect. Hypothesizing interdependence of genetic risk factors prompted us to perform exploratory studies applying the Ordered-Subset Analysis (OSA) approach using LOD scores as the trait covariate for ranking families. We employed OSA to test for interlocus correlations between loci with LOD scores ≥1.5, and empirically determined significance of linkage in optimal OSA subsets using permutation testing. Exploring phenotypic correlates as the basis for linkage increases involved comparison of mean scores for quantitative trait-based subsets of autism between optimal subsets and the remaining families.

**Results:**

A genome-wide screen for autism loci identified the best evidence for linkage to 17q11.2 and 19p13, with maximum multipoint heterogeneity LOD scores of 2.9 and 2.6, respectively. Suggestive linkage (LOD scores ≥1.5) at other loci included 3p, 6q, 7q, 12p, and 16p. OSA revealed *positive *correlations of linkage between the 19p locus and 17q, between 19p and 6q, and between 7q and 5p. While potential phenotypic correlates for these findings were not identified for the chromosome 7/5 combination, differences indicating more rapid achievement of "developmental milestones" was apparent in the chromosome 19 OSA-defined subsets for 17q and 6q. OSA was used to test the hypothesis that 19p linkage involved more rapid achievement of these milestones and it revealed significantly increased LOD* scores at 19p13.

**Conclusions:**

Our results further support 19p13 as harboring an autism susceptibility locus, confirm other linkage findings at 17q11.2, and demonstrate the need to analyze more discreet trait-based subsets of complex phenotypes to improve ability to detect genetic effects.

## Background

Autism (OMIM # 209850) is a neurobehavioral disorder involving deficits in language and social abilities and patterns of repetitive behaviors, restricted interests and resistance to change. The most recent estimate of population prevalence for the broader autism spectrum indicates a rate of 34/10,000 (~1/300) [1], with a male: female ratio of 4:1 [2,3]. Evidence from various studies indicates idiopathic autism has a complex genetic etiology. Twin studies show a concordance of 60% among monozygotic (MZ) twins and 0% among dizygotic (DZ) pairs for classic autism, but this increases to 92% for MZ pairs and 10% for DZ pairs when a broader phenotype of related social and language abnormalities is included [4,5]. The sibling recurrence risk is suggested to be ~3–10% but may be underestimated as a result of "stoppage rules" [6-8], and the relative risk is thus 30–100 times that in the general population [5,7]. Heritability is estimated at 90%, which is among the highest for psychiatric disorders. While the data do not strongly endorse any one model for inheritance, twin and family studies support a multilocus etiology with as many as 10–20 loci (reviewed in [9-11]).

Genome-wide screens of multiplex autism families for susceptibility loci [12-22] have identified a few genomic regions in common across multiple studies; 7q and 2q have received the greatest attention [17,19,20,23-28], with support from chromosomal abnormalities affecting these regions in idiopathic autism (reviewed in [29]). Genetic studies of autism are substantially complicated by clinical and locus heterogeneity, and it is possible that epistatic or epigenetic mechanisms may play important roles in genetic etiology [9,30]. Analytical strategies that address the latter concerns are limited, and most studies to date have focused on analysis of main effects using a global autism diagnosis to define affection status. Moving forward, more sophisticated approaches are being proposed in which trait-based subsets of the broader autism phenotype are used in genetic analyses. Similarly, given the interdependence of genes and their protein products within biological systems, analytical approaches that address potential interaction between susceptibility loci will also be critical to characterizing gene-phenotype relationships in autism.

We report a second generation 10-cM microsatellite-based genomic screen of multiplex autism families. The dataset for this screen includes 71 families recruited by the Tufts/New England Medical Center, a well-characterized set of 85 families from the Autism Genetics Resource Exchange (AGRE), and 2 families from Vanderbilt University. Several sites of suggestive linkage are identified, although none meet criteria for genome-wide significance. The loci with greatest support for linkage were 17q11.2 and 19p13; the latter site demonstrated significantly increased allele-sharing when the Ordered-Subset Analysis (OSA) algorithm was employed using a quantitative trait-based autism phenotypic subset related to specific "developmental milestones" as a covariate to rank families.

## Methods

### Sample and Demographics

The demographics for the 158 family dataset comprising the studies in this report are shown in Table 1. Families were recruited through three sites: (a) 71 families from the Tufts/NEMC site, (b) 2 families from the Vanderbilt University site, and (c) the remainder of families (85) were chosen from the AGRE repository based on our own recruitment criteria. Multiplex families (mostly affected sibling-pairs) had one affected individual who met full criteria for autistic disorder based on Autism Diagnostic Interview-Revised (ADI-R; [31-33])) algorithm scores, while the second individual either met criteria or in some cases was under the cut-off by only one or two points. Exclusion criteria included dysmorphic features, abnormal karyotype, diagnosis of fragile X syndrome, and other genetic disorders of known etiology. Individuals were assessed by the respective groups using the ADI-R at a developmental age >18 months; Tufts/NEMC and Vanderbilt groups included individuals between the ages of 4 and 22; in cases in which ADI-R interviews were performed initially at <4 years, they were repeated when the probands reached 4 years of age. All individuals were additionally assessed using the Autism Diagnostic Observation Schedule [32,34] and the Vineland Adaptive Behavior Scales – Interview Edition [35,36].

### Genotype data and statistical analysis

DNA from Tufts and Vanderbilt samples was obtained from peripheral blood or immortalized lymphoblastoid cell lines using the PureGene Kit (Gentra Systems). While a minority of families from the Tufts/NEMC cohort had been genotyped previously [13], both new and previously genotyped families were genotyped by deCODE (Reykjavik, Iceland) using their 500 marker (~8 cM intermarker spacing) panel and corresponding genetic map [37]. Genotype data were obtained from the AGRE website [38] for families whose samples were purchased from the AGRE repository and included in this study. Clinical procedures and genotyping for the AGRE sample has been described previously [18,39]. AGRE samples and corresponding genotype data had a distinct but overlapping panel of markers compared to the Tufts and Vanderbilt families. AGRE genetic markers were placed on the deCODE map, with order and spacing properly insured through exhaustive comparisons between genotyped markers, available genetic maps, and physical DNA sequence assemblies in both public and Celera databases.

Genotype data for each chromosome underwent thorough error detection and genotype confirmation. Initially, data were tested for Mendelian inconsistencies using PEDCHECK [40] and RELPAIR [41], followed by SIMWALK2 [42] for haplotype construction to detect genotyping errors reflected by unlikely double recombinants. In the event of a highly improbable genotype, the data for that marker were excluded for the family.

Allele frequencies were estimated using genotype data from all unrelated individuals in the combined dataset, consisting of more than 300 chromosomes. Allele frequencies were compared with available data from other Caucasian populations, and no significant differences were observed (data not shown). The LAPIS program of the PEDIGENE system [43] was used to output appropriate analysis files for the different programs.

Linkage was analyzed using both model-dependent and model-independent methods. For autosomes, two-point and multipoint heterogeneity LOD (HLOD) scores were calculated under both dominant and recessive models using Allegro [44]. Disease allele frequency was estimated to be 0.01 and 0.1 for dominant and recessive models, respectively. Phenotypic status was only considered for affected individuals, and other family members were designated as having an unknown phenotypic status. Nonparametric allele-sharing LOD* values were calculated using affected relative pair data based on an exponential model using the *S*_pairs _scoring function as recommended by McPeek [45]. NPL scores and corresponding P-values were also calculated by Allegro. Data from the X chromosome were analyzed using ASPEX [46] and FASTLINK [47] to calculate two-point and multipoint MLOD scores. Peak parametric (HLOD) or nonparametric LOD* scores ≥1.5 were considered as "suggestive" evidence for linkage and listed in Table 2, along with corresponding peak marker, deCODE cM location, and chromosomal band position.

The nonparametric genome-wide significance threshold [48,49] for linkage at the P = 0.05 level was determined by conducting simulations using Merlin [50] with the current dataset. The Simulate option in Merlin was used to produce 1000 random datasets that preserve the properties of the original data for marker informativeness, spacing and missing data patterns. An empirical significance threshold was determined by using the 95^th ^percentile of the resulting distribution.

OSA [51] identifies genetically more homogeneous subsets of the overall data by ordering families according to covariate trait values in ascending or descending order. OSA takes the first family and calculates an allele-sharing LOD* score. In an iterative process, OSA successively adds families, re-calculating LOD* scores with each addition, and it identifies the division in the dataset at which maximum linkage is obtained on the chromosome being analyzed. Permutation testing is used to determine the empirical significance of the observed results. OSA has been applied with success to identify or increase evidence in support of linkage to complex disease susceptibility loci [52-54].

To explore potential genetic interaction or other genetic correlations between sites of main effect (i.e. suggestive linkage), OSA was applied using family-specific LOD scores as the covariate trait. Families were ranked according to the family-specific LOD score at peak sites demonstrating LOD scores ≥1.5. Allele-sharing analysis was performed for the other six chromosomes using the OSA algorithm. For instances of empirically significant increases in evidence for linkage, we explored the nature of the genetic correlation to ask whether it reflected clinical correlations in the respective subsets. We employed ADI-based factor subsets, identified by principal components analyses of ADI/ADI-R items, to represent putative phenotypic subsets in autism [55,56]. The ADI-based variable clusters correspond to "(1) language, (2) social intent, (3) developmental milestones, (4) rigid-compulsive behaviors, (5) savant skills, and (6) sensory aversion", as determined by Folstein and colleagues [55]; and (7) "insistence on sameness" as described by Cuccaro and colleagues [56]. We thus compared the seven ADI-based factor score means (both the mean of family means and the mean of affected individuals) using a t-test for the families above and below the OSA-determined split in the dataset resulting in maximal linkage. Subsequent analysis involved specific examination of the "developmental milestones" cluster. The milestones factor indexes on the following ADI items: "*(1) To walk unaided; (2) to sit unaided on flat surface; (3) age of first single words; (4) age of first phrase; (5–6) acquisition of bladder control: daytime, night; (7) acquisition of bowel control.*"

Analysis of the "developmental milestones" factor as a potential phenotypic subset related to the autism linkage correlations was performed by applying the OSA algorithm. We used "developmental milestones" family means, normalized via SAS and Box-Cox transformation procedures, as an ascending ranking covariate. LOD* scores were calculated according to the OSA algorithm, and the resulting increase in linkage achieved with the OSA-determined family subset was analyzed through permutation testing.

Approval for these studies was granted by the respective Institutional Review Boards at Tufts University School of Medicine/New England Medical Center and Vanderbilt University Medical Center. Additionally, all studies were performed with informed consent provided by the families participating in the research.

## Results

**Figure 1:**
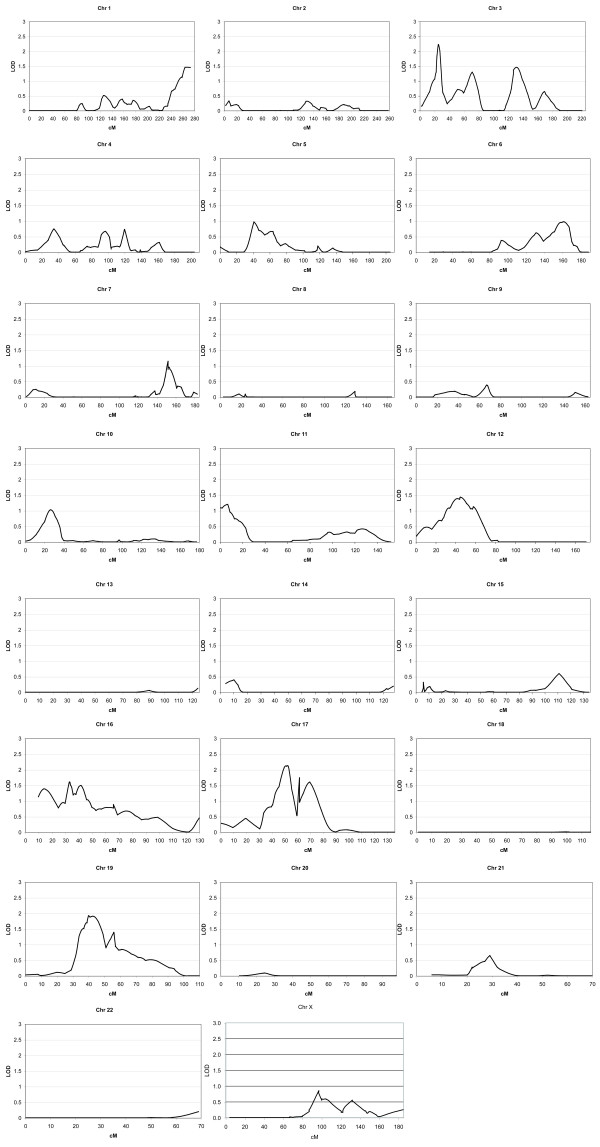
**Genome-wide nonparametric linkage analysis in 158 multiplex families for autism loci. **Individual plots show allele-sharing LOD* scores calculated for autosomes using Allegro and MLOD scores for the X chromosome calculated using ASPEX.

Seven chromosomes revealed one or more regions of linkage with a model-dependent or model-independent LOD score ≥1.5 (Figure 1). No locus reached the empirically derived genome-wide significance level of 2.92. These suggestive loci include 3p25, 6q23, 12p12, 16p12-p13, 17q11, 17q21 and 19p13 (Table 2). Data provide the most compelling support for 17q11.2 and 19p13 as harboring autism susceptibility loci.

**Figure 2:**
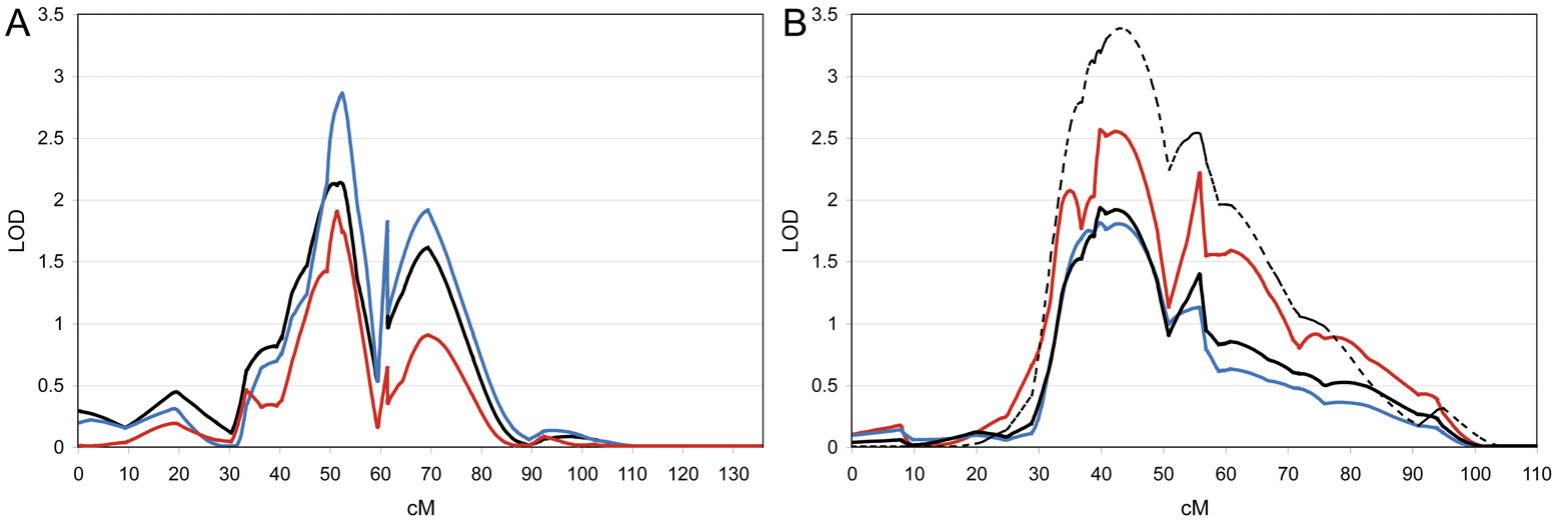
**Multipoint linkage analysis under all models for chromosomes 17 (A) and 19 (B). **Multipoint parametric HLOD plots for both dominant (blue) and recessive (red) models, and nonparametric allele-sharing LOD* values (black) are displayed across the respective chromosomes. OSA analysis using ascending "developmental milestones" factor scores to order families is shown for chromosome 19, for which a 92-family optimal subset was identified and used to calculate allele-sharing LOD* scores (dashed black line).

For 17q11.2, peak linkage was observed at 53 cM, corresponding to marker D17S1294 (Table 2), at which we see a multipoint HLOD of 2.85. Nonparametric multipoint analysis revealed an allele-sharing LOD* score of 2.13 and an NPL score of 2.84 (P = 0.0024). A second telomeric peak can be distinguished on 17 at ~69 cM, corresponding to 17q21.2. Marker D17S1299 at this site yielded a HLOD of 1.9, a LOD* of 1.66 and an NPL score of 2.26 (P = 0.012). The more proximal peak at ~53 cM lies in close proximity (~150 kb) to the serotonin transporter (*SLC6A4*) locus, long considered to be an attractive functional candidate gene for autism and other neuropsychiatric conditions. Figure 2 shows multipoint LOD score plots for both dominant and recessive parametric (HLOD) and nonparametric allele-sharing LOD* values for chromosomes 17 and 19.

The second most significant result was observed on 19p13, where peak linkage was detected at marker D19S930, revealing a multipoint HLOD of 2.55 at ~40 cM (Table 2 and Figure 2). Nonparametric analyses detected a LOD* of 1.92 and a corresponding NPL of 2.77 (P = 0.003). As with chromosome 17, the multipoint analyses show a second more telomeric peak, corresponding to marker D19S113. The recessive HLOD at this site was 2.20, with model-independent LOD* and NPL values of 1.39 and 2.10 (P = 0.018), respectively.

**Figure 3:**
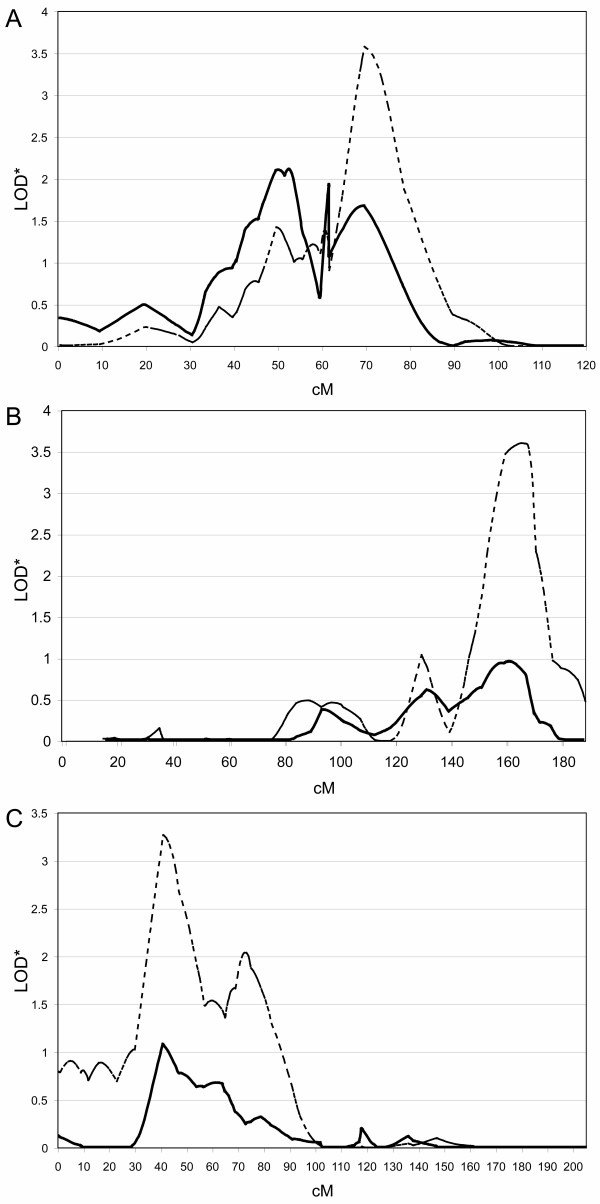
**OSA using family-specific LOD scores as the ranking covariate. **Families were ordered based on descending LOD scores at peak linkage for 19p13 and allele-sharing LOD scores calculated in the optimal subset for (A) chromosome 17 or (B) chromosome 6. Families were also ranked based on descending LOD scores at peak linkage on chromosome 7q (C), and LOD scores calculated for chromosome 5. Solid lines reflect multipoint LOD scores corresponding to the entire dataset for the chromosome being analyzed, while dashed lines represent analysis of the optimal subset (above the dataset division in all cases) identified from OSA; these were 52 families for chromosome 17, 30 for chromosome 6 and 41 families for chromosome 7.

To address the possibility of gene-gene interaction, we applied the OSA approach with family-specific LOD scores as the ranking trait. Families, almost all of which are affected sib-pairs, were ranked in both ascending and descending order using family-specific LOD scores. The three most significant correlations are presented in Figure 3. Using chromosome 19 LOD scores as the covariate, the results on chromosome 17q, while non-significant (P = 0.1), showed an increase in linkage at the more distal peak on 17q21.1 from a LOD* of 1.7 to 3.6 and identified an optimal subset of 52 families. Applying the same covariate, a significant increase was seen on chromosome 6q, with a smaller, completely overlapping, 30-family optimal subset. This subset resulted in an increase in LOD* values from 1.0 to 3.6 at ~164 cM (P = 0.004). Another significant finding involves the 7q region, possibly representing the most replicated site of linkage in autism (reviewed in [9,29]). Given a substantial focus on this region over several years, we lessened our criteria to examine any other chromosome demonstrating a LOD score >1. Application of OSA using chromosome 7q linkage data, again ranking families based on LOD scores in a descending manner, lead to a significant increase in linkage on 5p at ~41 cM from a LOD* of 1.1 to 3.3 in a 41-family subset. Thus, in these three cases, notwithstanding the nonsignificance of the 19p13/17q21 result, there is a *positive *correlation of linkage in varying but overlapping subsets of the data between these respective pair-wise locus combinations.

To further explore the basis of the observed results, we tested the hypothesis that underlying phenotypic correlates might explain genetic correlations. We compared the mean values for the seven factor traits in the optimal subsets compared to the means of the remaining families using a t-test, both under assumption of equal and unequal variances. This comparison for all seven available factors revealed a nominally significant differences in the chromosome 19 optimal subsets identified from OSA analysis of chromosomes 17 (52 families) and 6 (30 families) for the "developmental milestones" cluster. The families in the optimal OSA subset have lower scores and therefore are more rapidly achieving developmental milestones. A similar procedure for the chromosome 7-based subset revealed no obvious differences in any of the factors (data not shown).

To directly test the hypothesis that chromosome 19 linkage was related to reduced affection for the "developmental milestones" factor, we performed an OSA analysis in which families were ranked in ascending order based on mean values for the milestones factor score. Figure 2 shows the results from this analysis, which generated increased evidence for linkage to 19p13 with peak LOD* scores increasing from 1.9 to 3.4. Permutation testing revealed this increase to be empirically significant (P = 0.04), thus further supporting 19p13 as harboring a genetic risk factor for autism.

## Discussion

We have presented evidence in support of autism susceptibility loci on chromosomes 17q and 19p. Our results suggest that the 19p locus is related to a phenotypic profile involving a more rapid achievement of particular "developmental milestones". Features indexed in this ADI-based factor are: (1) ability to walk unaided; (2) ability to sit unaided on a flat surface; (3) age of first single words; (4) age of first phrase; (5–6) acquisition of bladder control: daytime and night; and (7) acquisition of bowel control. Analyses leading to this conclusion also showed positive genetic correlations between optimal OSA-defined subsets contributing to linkage at 19p13 and increases in linkage at loci on 17q21 and 6q23. A similar positive genetic correlation was shown for chromosomes 7q and 5p, however this observation lacks evidence of an underlying phenotypic relationship based on available ADI variable clusters. While the increase in linkage at 17q21 was not empirically significant, the differences in "milestone" score means between the optimal chromosome 19 subsets seen for both chromosomes 17 (52 families) and 6q (30 families) were significant. These exploratory data led to the significant finding of increased linkage in the single direct test of our hypothesis concerning the phenotypic correlation related to chromosome 19 linkage.

Despite the significance of the final results on 19, we remain cautious in the interpretation of the overall results. As with a number of other genomic screens in autism, no single main effect locus achieved genome-wide significance. Support for a number of these loci, particularly at 17q11.2 and 19p13 comes from similar suggestive linkage in other genomic screens for autism. Although not all screens detect these loci (not an uncommon finding in linkage studies for complex genetic disorders), the evidence is strong regarding an effect at 19p, within 10 cM of our peak: (1) Shao et al reported an MMLS = 1.21 and an MLOD = 1.38 [14]; (2) the Paris Autism Research International Sibpair Study (PARIS) an MMLS = 1.37 [12]; the International Molecular Genetic Study of Autism Consortium (IMGSAC) reported an MLS of 1.16 [15]; the Mt. Sinai group reported an NPL of 1.56 which increased to 2.31 when only families with obsessive-compulsive behaviors were considered for this region [22].

Similarly, several groups have reported evidence for linkage at 17q11. The recently published AGRE follow-up genomic screen identified an MLS of 2.83 near *SLC6A4 *[21]. A genome scan for attention deficit/hyperactivity disorder (ADHD) identified an MLS of 2.98 near this locus [57]. An IMGSAC follow-up screen for autism [27] reported a maximum multipoint LOD score of 2.34 at HTTINT2 in the *SLC6A4 *gene on chromosome 17q11.2. Our own more preliminary analysis of linkage in this region with a highly overlapping dataset to that in the current study, revealed very similar results [58]. Given our inclusion of some AGRE families, it is not completely unexpected that 17q11.2 linkage is similar to that seen the larger AGRE 2^nd^-stage screen [21], however AGRE families only represented about half of the overall dataset. Families recruited from the Tufts/NEMC site are clearly contributing to this linkage based on the LOD score-based optimal family subset compositions.

The 17q21 locus is worth further consideration. Our data support the premise that the adjacent linkage peaks represent distinct loci and are not an artifact of primary linkage at 17q11.2. The evidence for linkage at 17q21, while weaker than that at 17q11.2 only 16 cM centromeric, specifically showed an, albeit nonsignificant, interlocus correlation with 19p13 linkage. Linkage at 17q11.2 in this subset of families actually decreases slightly. Of particular interest is the fact that the distal region harbors the integrin β3 (*ITGB3*) locus, which was identified recently from a genome-wide quantitative trait locus (QTL) association screen for platelet serotonin levels [59]. We see nominal evidence of linkage to autism at this site, and ~20–25% of individuals with autism have elevated levels of circulating serotonin.

The other "suggestive" (LOD ≥ 1.5) loci reported here have also been detected in other genome-wide scans for autism loci. A broad region of 7q has been detected in most screens [17,20,23,25,27,28]. The 16p region has been identified by IMGSAC, and others [15,18,22,27]. Chromosomal abnormalities have also been reported for several of these regions in cases of autism (reviewed in [11]). Linkage at 3p was reported by at least two groups [14,17]. Linkage has also been reported at our 6q locus by at least one other group [12]. Thus, while not significant, the replication of these linkage observations provides support for the likelihood that many of these loci represent true sites of main effect in autism.

The application of OSA to detect putative interlocus correlations between the 19p13 and 17q21, 19p13 and 6q23, and between 7q35 and 5p are limited to some degree in significance by their highly exploratory and hypothesis-generating nature. Given the number of comparisons between loci, and the number of comparisons between optimal subset pairs (on 19p or 7q) for the traits means, the potential for type I error is increased. Therefore our interpretation must be cast alongside appropriate caveats. Nevertheless, the multiple exploratory comparisons generated a hypothesis: *that linkage to 19p13 was related to a more rapid achievement for specific milestones*. We tested this hypothesis with a single analysis revealing an empirically significant increase in linkage at 19p13. Our results of autism linkage and its increase using an ascending milestone score covariate in OSA, taken in the context of replicated observations of suggestive linkage by other groups, strengthens support for the presence of an autism gene at this site. In the end, ultimate interpretation will rely upon replication of these phenomena with independent samples to confirm these observations.

Finally, our results highlight the utility of using trait-based subsets of autism to identify putative susceptibility loci for this complex disorder. We and others have hypothesized a likely increased specificity of individual risk genes and corresponding alleles for traits or subphenotypes comprising the broader autism spectrum. Therefore methods such as OSA with power to identify more homogeneous samples and QTL (quantitative trait locus) linkage and association analyses should provide greater sensitivity in the discovery of disease genes in the context of locus and clinical heterogeneity. Additionally, OSA or other forms of conditional linkage analyses, have the ability to uncover potential interactions between loci, an important concept since the inherent interdependence of proteins in common pathways or networks acting during development and normal neuronal function could be easily imagined to act genetically in concert with one another.

## Conclusions

We report evidence to support linkage of autism to 17p11.2 and 19p13. Exploratory analyses to test for correlations between suggestively-linked loci, using the OSA method revealed positive correlations of linkage (i.e. in overlapping families) between 7q and 5p, 19p and 6q, and possibly 19p and 17q22, distal to peak linkage at 17q11.2. Comparing mean scores for ADI-derived factor traits from families above and below the OSA-defined split maximizing linkage, suggested a positive correlation between 19p13 linkage and a more rapid achievement of "developmental milestones" as measured by items in this cluster of ADI variables. We tested this hypothesis by applying OSA with descending "developmental milestone" scores as the ranking covariate, and detected an empirically-significant increase in linkage to 19p13. These findings further support evidence for an autism susceptibility locus in 19p13 and underscore the utility in applying trait subsets in complex disorders to identify genetic risk factors.

## Competing interests

The authors declare that they have no competing interests.

## Authors' contributions

JLM and LMO were responsible for conducting the genome-wide linkage analyses; JLM conducted all OSA analyses, was largely responsible for coordinating and executing the bulk of the reported studies, was key to drafting and editing the manuscript text, and in preparing the figures. CL and JLH provided input into the design and interpretation of statistical results, and provided guidance for conducting the genome-wide simulations required to determine the significance threshold. JLH established the Core-based infrastructure in the Center for Human Genetics Research that facilitated this work and provided very helpful input into the development of the manuscript. SEF developed, with her group, the ADI-based clusters so crucial to examining phenotypic subsets in this paper, which she helped to edit. SEF was responsible for overseeing recruitment and the phenotypic assessment of families from her research group, then at Tufts/NEMC. GC is the clinical coordinator at the Vanderbilt site and oversees ascertainment, recruitment and detailed phenotypic assessment of affected individuals. KG is the Vanderbilt data coordinator and is responsible for management and oversight of family information, pedigree data, and status of DNA, blood or cell line samples for family members. She is directly responsible for preparation of Table 1 of this paper. JSS and JLH are Principal Investigator (PI) and co-PI, respectively, of the current study, and together conceived of and implemented the experimental strategy in close consultation with all other co-authors. JSS initially drafted the manuscript and with JLM incorporated changes suggested by co-authors. JSS was responsible for preparation of all final versions of figures from earlier versions provided by co-authors.

## Pre-publication history

The pre-publication history for this paper can be accessed here:



## Figures and Tables

**Table 1:** Sample Demographics

**Families in Linkage Screen**	**158**
Tufts/NEMC	71
AGRE	85
Vanderbilt	2

**Affected Individuals**	**333**
Males	257
Females	76

**Tufts/NEMC Total**	**148**
Males	117
Females	31
**AGRE Total**	**181**
Males	137
Females	44
**Vanderbilt Total**	**4**
Males	3
Females	1

**Age at ADI (range)**	**2–46.7**

Tufts/NEMC*	2–46.7
AGRE	2–38.0
Vanderbilt	6.2–9.2

**Ethnicity (Individuals)**	

**Caucasian (242)**	**73.0%**
Tufts/NEMC (130)	87.8%
AGRE (108)	59.7%
Vanderbilt (4)	100.0%

**Hispanic-Latino (14)**	**4.2%**
AGRE (14)	7.7%

**African-American (8)**	**2.4%**
Tufts/NEMC (6)	4.1%
AGRE (2)	1.1%

**Asian (8)**	**2.4%**
Tufts/NEMC (2)	1.4%
AGRE (6)	3.3%

**Multi-ethnic (14)**	**4.2%**
AGRE (14)	7.7%

**Other (2)**	**0.6%**
Tufts/NEMC (2)	1.4%

**Unknown (45)**	13.5%
Tufts/NEMC (8)	5.4%
AGRE (37)	20.4%

**I.Q. estimate distributions**	

Tufts/NEMC** (148)	
<30	13
30–49	21
50–69	37
70+	17
Unknown	60

AGRE*** (181)	
<30	17
30–49	27
50–69	15
70+	13
Unknown	109

Vanderbilt*** (4)	
<30	1
30–49	1
50–69	0
70+	2
Unknown	0

*ADI-Rs performed <4 yrs were repeated at 4 yrs for Tufts/NEMC families

**IQ estimates are based on the Vineland Daily Living standard scores

***IQ estimates are based on overall Vineland Adaptive Behavior standard scores

**Table 2:** Linkage Data for Loci with LOD Scores >1.5

**Chromosome**	**deCODE cM**	**Marker**	**HLOD**	LOD*
3p25.3	25	D3S3691	1.76^R^	**2.22**
6q23.2	131	D6S1656	**1.61^R^**	0.62
7q35	152	D7S2195	**1.65^R^**	1.14
12p12.1	45	D12S1591	**1.50^R^**	1.43
16p13.2	15	ATA41E04	**1.64^D^**	1.38
16p13.12	33	D16S3062	**1.87^D^**	1.60
16p12.3	43	D16S490	**1.80^D^**	1.49
17q11.2	53	D17S1294	**2.85^D^**	2.13
17q21.2	69	D17S1299	**1.90^D^**	1.60
19p13.11	40	D19S930	**2.55^R^**	1.92
19p13.11	56	D19S113	**2.20^R^**	1.39

R: recessive; D: dominant
